# The stability of blood Eosinophils in chronic obstructive pulmonary disease

**DOI:** 10.1186/s12931-020-1279-4

**Published:** 2020-01-10

**Authors:** Gabriella H. Long, Thomas Southworth, Umme Kolsum, Gavin C. Donaldson, Jadwiga A. Wedzicha, Christopher E. Brightling, Dave Singh

**Affiliations:** 1 Division of Infection, Immunity and Respiratory Medicine, School of Biological Sciences, Faculty of Biology, Medicine and Health, Manchester Academic Health Science Centre, The University of Manchester, Manchester, UK; 2 Medicines Evaluation Unit, Manchester University NHS Foundation Trust, Manchester, UK; 3 National Heart and Lung Institute, Imperial College London, London, UK; 4 Institute for Lung Health, University of Leicester, Leicester, UK

**Keywords:** COPD, Eosinophil, Airway inflammation

## Abstract

Blood eosinophils are a predictive biomarker of inhaled corticosteroid response in chronic obstructive pulmonary disease (COPD). We investigated blood eosinophil stability over 1 year using the Global Initiative for Chronic Obstructive Lung Disease (GOLD) 2019 thresholds of < 100, 100- < 300 and ≥ 300 eosinophils/μL in 225 patients from the COPDMAP cohort. Blood eosinophils showed good stability (rho: 0.71, *p* < 0.001, ICC 0.84), and 69.3% of patients remained in the same eosinophil category at 1 year. 85.3% of patients with eosinophils < 100 cells/μL had stable counts. The majority of blood eosinophil counts remain stable over 1 year using the GOLD 2019 thresholds.

## Introduction

Randomised controlled trials have shown a relationship between blood eosinophil counts and the effects of inhaled corticosteroid (ICS) on exacerbations in chronic obstructive pulmonary disease (COPD) [1–3]. Data modelling suggests that ICS effects are observable at > 100 cells/μL, with the greatest effects at ≥300 cells/μL [4]. The Global Initiative for Chronic Obstructive Lung Disease (GOLD) 2019 report recommends < 100 and ≥ 300 eosinophils/μL thresholds to identify patients who are least and most likely, respectively, to benefit from ICS treatment [3].

The stability of blood eosinophil counts over time is an issue that is relevant for the use of this biomarker in COPD patients. Changes in blood eosinophil counts that result in movement across the thresholds recommended by GOLD may influence pharmacological treatment decisions. Greater variability for repeated blood eosinophil counts has been reported at higher eosinophil levels [5–7], but the stability of eosinophil counts has not been assessed using current GOLD recommended thresholds. We have analysed the stability of blood eosinophil counts over 1 year in the COPDMAP observational cohort study (www.copdmap.org; study design described previously [8]) using the GOLD 2019 thresholds.

## Methods

COPD patients with two blood eosinophil measurements taken at baseline and 12 months apart during stable state were included (*n* = 225) to determine within-subject variability over time. Patients with a previous asthma history or requiring maintenance oral corticosteroid therapy were excluded from this analysis. All patients provided written informed consent and local ethical approvals were obtained (11/L0/1630;10/H/1003/108; 07/H0406/157). Additional methods are in the online supplement (Additional file 1). Patients were subdivided into 3 groups using the baseline blood eosinophil count; < 100, 100- < 300 or ≥ 300 cells/μL. Data were analysed using spearman’s rank test (Prism 7.0, GraphPad, USA), intraclass correlation coefficient (ICC) of log-transformed data, analysis of variance (ANOVA), linear regression, Bland-Altman and repeatability coefficient analysis (RCA) calculated as 1.96 multiplied by $\sqrt{2}$ times the within-subject standard deviation (SPSS 22.0, IBM, Armonk, USA) [9]. ICC values are interpreted as excellent (*>* 0.75), fair to good (0.40–0.75), or poor (*<* 0.40) [9]. *P* < 0.05 was considered statistically significant.

## Results

Patients had a mean (SD) age of 69.8 (8.4) years, forced expiratory volume in 1 s (FEV_1_) 55.9% (18.2%) predicted, COPD assessment test (CAT) score 16.9 (7.5), and 1.6 (2.1) exacerbations per year; 67.1% were male, 29.3% were current smokers and 80.4% were on ICS therapy. The number of patients with blood eosinophils < 100, 100- < 300 and ≥ 300 cells/μL were 38 (16.9%), 139 (61.8%) and 48 (21.3%) respectively; clinical characteristics of these groups were similar.

**Fig. 1:**
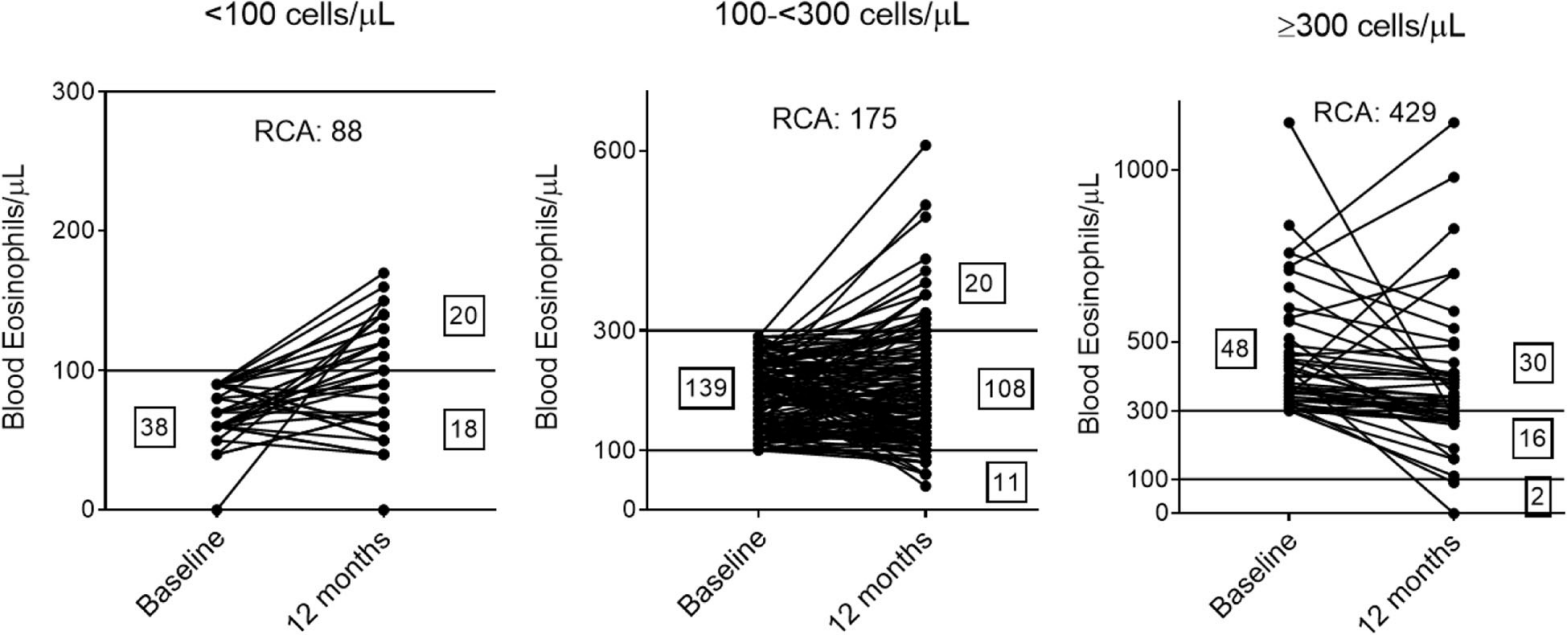
Stability of blood eosinophils stratified by baseline blood eosinophils < 100, 100–300 or ≥ 300 cells/μL. Number of patients are displayed in boxes. Repeatability coefficients (RCA) were 88, 175 and 429 cells/μL, respectively

There was a significant correlation between blood eosinophil counts at baseline and 12 months (*n* = 225); rho = 0.71, *p* < 0.001. The ICC value was 0.84, indicating excellent repeatability. Bland-Altman regression analysis showed lowest and greatest variability at < 100 eosinophils/μL and ≥ 300 eosinophils/μL at baseline respectively (linear regression p < 0.001). Repeatability coefficients for blood eosinophils < 100, 100- < 300, and ≥ 300 cells/μL showed that 95% of measurements at 12 months were within 88, 175 and 429 cells/μL of the baseline value, respectively [Fig. 1]. 69.3% (*n* = 156) of patients had blood eosinophils that remained within the same category over 12 months; 47.4, 77.7 and 62.5% of patients remained stable at < 100, 100- < 300 cells/μL, and ≥ 300 cells/μL, respectively [Fig. 1]. Of those patients with blood eosinophils < 100 or ≥ 300 cells/μL who changed group, the majority (36/38 patients) moved to the adjacent group.

**Fig. 2:**
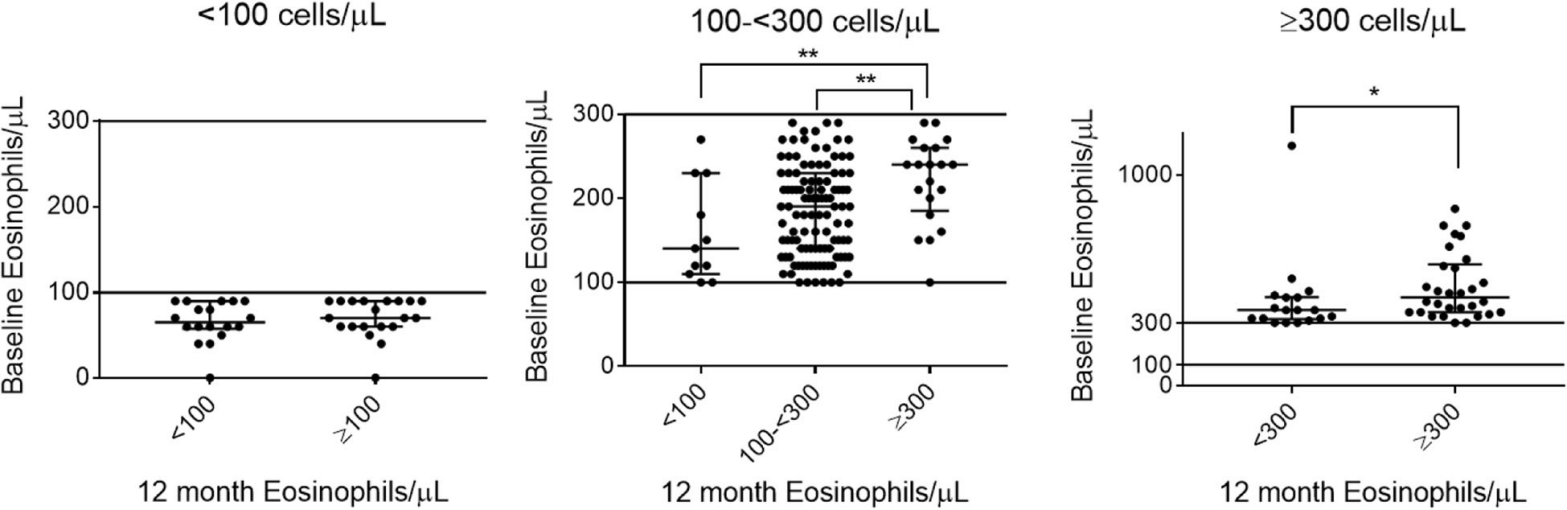
Comparison of baseline blood eosinophil counts in those who remained versus changed 12-month blood eosinophil group. Graphs stratified by baseline groups < 100, 100- < 300 or ≥ 300 cells/μL, respectively. Error bars represent median and IQR. * = *p* < 0.05. ** = *p* < 0.01

Baseline blood eosinophil counts were similar in patients who remained < 100 cells/μL compared to those who changed group [Fig. 2]. Blood eosinophil counts in patients who remained in the 100- < 300 cells/μL group were lower compared to those who changed to ≥ 300 cells/μL (medians 190 vs 240 cells/μL, respectively, *p* = 0.0053), while the lowest counts were observed in those who moved to < 100 cells/μL (median 140 cells/μL, *p* = 0.12 for comparison versus patients who remained in the 100- < 300 cells/μL group). For patients with blood eosinophils ≥ 300 cells/μL, those that moved below 300 cells/μL at 12 months had a lower baseline measurement compared to those who remained ≥ 300 cells/μL (median 360 vs 420, respectively, *p* = 0.024). Only 3 patients (out of 225) changed ICS use within the 12-month observation period; these patients were commenced on ICS treatment, and none dropped to a lower eosinophil group.

There were 99 patients who experienced ≥1 exacerbation within the 12 month period between stable state blood eosinophil measurements. The correlation between repeated blood eosinophil measurements was similar in patients with ≥1 exacerbation (rho = 0.73, *p* < 0.001) and those with no exacerbations (*n* = 126; rho = 0.71, *p* < 0.001). For patients with ≥1 exacerbation, 72.7% remained within the same group, whilst 66.7% of those with no exacerbations were stable over 12 months. Similar results were obtained when patients with exacerbations were divided into those with 1 exacerbation within the 12-month period (*n* = 64; blood eosinophils were stable in 71.9%) and those with ≥2 exacerbations (*n* = 35; blood eosinophils were stable in 74.3%).

Subgroup analysis of blood eosinophil stability according to ICS use showed similar results in those using ICS (*n* = 181; rho = 0.58, *p* < 0.001) and those not using ICS (*n* = 44; rho = 0.47, *p* = 0.001). For patients using on ICS, 70.2% had blood eosinophil counts that remained in the same group over 12 months, while this was 65.9% for those not using ICS.

## Discussion

The main findings were that (1) blood eosinophil stability over 12-months was excellent when analysed by ICC (0.84), (2) regression analysis showed greater variability at higher eosinophil counts (3) 69.3% of patients remained stable within the same category (4) movement between groups was more likely when baseline values were closer to a threshold. The main clinical usefulness of these findings is to demonstrate that the majority of COPD patients remain within the same blood eosinophil category (defined by GOLD) after 1 year. This provides reassurance regarding treatment decisions using this biomarker.

GOLD 2020 now states that the thresholds of < 100 cells/μL and > 300 cells/μL are “estimates, rather than precise cut-off values” with respect to the prediction of ICS effects [10]. This concept is important when considering movement across these thresholds after repeat testing, which occurred in approximately 30% of patients in this study. The continuous nature of the blood eosinophil count – ICS response relationship means that small numerical changes after a repeat test are unlikely to be associated with a significant change in ICS responsiveness. We observed that movement across a threshold was more likely for baseline measurements that were closer to the threshold; this appears to be natural measurement variation across a threshold rather than a significant change in the pathophysiology of an individual. We suggest that these small numerical movements across a threshold should not change the original clinical interpretation based on the baseline blood eosinophil count. Similarly, 1-year stability in COPD patients (*n* = 27,557) at 12-months has been reported (ICC = 0.7), with lower blood eosinophil reliability for measurements close to thresholds [11]. Average blood eosinophil counts for repeated measurements in clinical practice may overcome this variability [11].

Previous studies have shown similar ICC values for blood eosinophil counts [6, 7, 11]. E.g. Southworth et al. studied a smaller COPD cohort (*n* = 82) using thresholds < 150, 150- < 300 and ≥ 300 cells/μL; ICC = 0.89 and 0.87 at 6 months and 2 years respectively, with 71 and 64% respectively of patients remaining within the same category [6]. Greulich et al. (*n* = 334) similarly found those with blood eosinophils < 150 cells/μL had greater stability over 18 months [12]. The importance of our results is that we have used the thresholds defined by GOLD. Furthermore, we observed that 192/225 (85.3%) patients had eosinophil measurements that were stable using the 100 eosinophils/μL threshold. Similarly, Southworth et al. reported 91 and 85% stability at 6 months and > 2 years respectively using 100 eosinophils/μL [6]. Using only the lower eosinophil threshold in the GOLD report therefore appears to provide stable results in the majority of cases.

More patients moved across the threshold from the < 100 cells/μL group (52.6%) at baseline compared to the ≥ 300 cells/μL group (37.5%). Most of this variation from the < 100 cells/μL group (*n* = 36/38) resulted in movement to the adjacent group (100- < 300 cells/μL). The discordance between variability of absolute eosinophil numbers and movement across a threshold can be attributed to the wider measurement range at ≥ 300 cells/μL e.g. 20% (6/30) of individuals who remained ≥ 300 cells/μL had an absolute change ≥ 200 cells/μL, while all patients (*n* = 18) who remained < 100 cells/μL changed < 50 cells/μL.

Increased blood eosinophil counts in a subset of COPD patients has been demonstrated even when individuals with asthma or allergy are excluded [13]. The mechanism causing higher blood eosinophil counts in a subset of COPD patients is unclear. Blood eosinophils can be raised in the context of parasitic infections, but this was unlikely to be the cause in this UK based cohort study [14].

There was a high percentage of ICS use in our study population (80.4%), likely due to patients being recruited across 3 hospital based research centres rather than primary care. It would be interesting to see similar data in patients in primary care, with lower exacerbation risk and ICS use. Our data had a 1 year follow up, and future analyses could extend stability analysis to longer time periods.

In conclusion, approximately 70% of blood eosinophil measurements remained in the same category over 1 year using the GOLD 2019 thresholds. Small numerical changes may cause movement across a threshold.

## Supplementary information

**Additional file 1.** Additional Methods.

## Data Availability

All data generated or analysed during this study are included in this published article [and its supplementary information files].

## Abbreviations

ANOVA: Analysis of Variance
CAT: COPD assessment test
COPD: Chronic obstructive pulmonary disease
FEV_1_: Forced expiratory volume in 1 s
GOLD: Global Initiative for Chronic Obstructive Lung Disease
ICC: Interclass correlation coefficient
ICS: Inhaled corticosteroids
RCA: Repeatability coefficient analysis
