# Supplementary material for: The stability of blood Eosinophils in chronic obstructive pulmonary disease
Source: Respir Res. 2020 Jan 10;21:15. doi: 10.1186/s12931-020-1279-4 (PMC6954589; doi:10.1186/s12931-020-1279-4)
Supplement: Supplementary file 1 — Additional file 1. Additional Methods. [file 12931_2020_1279_MOESM1_ESM.docx]

**Supplement 1: Methods**

The COPDMAP prospective observational cohort study ([www.copdmap.org](http://www.copdmap.org)) recruited patients from 3 sites (Manchester, Leicester and London). The study was approved by local Research Ethics committees at each site (11/L0/1630; 10/H/1003/108; 07/H0406/157). All participants provided written informed consent prior to recruitment. Subjects aged 40 years or over had a physician diagnosis of COPD with a post-bronchodilator forced expiratory volume in 1 second (FEV_1_)/forced vital capacity (FVC) ratio <0.7 and ≥10 pack year smoking history. Included subjects were recruited between 2011 and 2014, and followed up for a minimum of 12 months. Full protocol summary is available at https://clinicaltrials.gov/ (NCT01620645). Demographic details were collected at the baseline visit. Symptoms were assessed using the modified medical research council (MRC) Dyspnoea Scale and COPD assessment test (CAT). Health-related quality of life was measured using the St George’s Respiratory Questionnaire (SGRQ). Lung function was assessed using spirometry performed in accordance with European Respiratory Society (ERS) and American Thoracic Society (ATS) guidelines[1]. Additional baseline variables collected included inhaled corticosteroid use and exacerbation history.

**Reference**

[1] Miller MR, Hankinson J, Brusasco V, Burgos F, Casaburi R, Coates A, et al. Standardisation of spirometry. Eur Respir J [Internet]. 2005 Aug 1;26(2):319 LP-338. Available from: http://erj.ersjournals.com/content/26/2/319.abstract
